# Minimal Invasive Navigation–Assisted Removal of Penetrating Metallic Foreign Bodies in the Craniomaxillofacial Region: A Case Report

**DOI:** 10.1155/crid/3914967

**Published:** 2026-05-22

**Authors:** H. Moghaddasi, P. Farnia, A. Ahmadian, N. Nadjmi, A. Parhiz

**Affiliations:** ^1^ Department of Medical Physics and Biomedical Engineering, Faculty of Medicine, Tehran University of Medical Sciences (TUMS), Tehran, Iran, tums.ac.ir; ^2^ Image-Guided Surgery Laboratory, Research Centre of Biomedical Technology and Robotics (RCBTR), Advanced Medical Technologies and Equipment Institute (AMTEI), Tehran University of Medical Sciences, Tehran, Iran, tums.ac.ir; ^3^ Research Centre of Intelligent Technologies in Medicine (RCITM), Advanced Medical Technologies and Equipment Institute (AMTEI), Tehran University of Medical Sciences (TUMS), Tehran, Iran, tums.ac.ir; ^4^ ZMACK association Cranio-Maxillofacial Surgery, AZ Monica Hospital Antwerp, Antwerp, Belgium; ^5^ Department of Cranio-Maxillofacial Surgery, Antwerp University Hospital, Edegem, Belgium, uza.be; ^6^ Faculty of Medicine & Health Sciences, University of Antwerp, Antwerp, Belgium, uantwerpen.be; ^7^ Craniomaxillofacial Research Centre, Tehran University of Medical Sciences (TUMS), Tehran, Iran, tums.ac.ir; ^8^ Department of Oral and Maxillofacial Surgery, School of Dentistry, Tehran University of Medical Sciences (TUMS), Tehran, Iran, tums.ac.ir

**Keywords:** craniomaxillofacial surgery, foreign body, image-guided navigation system

## Abstract

Removal of deeply embedded foreign bodies (FBs) in the craniomaxillofacial (CMF) region remains a significant surgical challenge because of the complex anatomy, proximity of critical neurovascular structures, and the limited accuracy of conventional imaging modalities for precise intraoperative localization. Inaccurate localization may increase operative time and the risk of iatrogenic injury. In recent years, there has been a considerable increase in the use of three‐dimensional image‐guided navigation systems (3D‐IGNS) in various maxillofacial procedures, especially the extraction of penetrating FBs in the CMF area. This report describes the clinical application of 3D‐IGNS for the safe and accurate removal of penetrating FBs in the CMF region. We present a case report of three patients with retained FBs resulting from gunshot injuries who underwent surgical extraction using 3D‐IGNS. Preoperative computed tomography imaging was used for navigation planning, allowing accurate real‐time intraoperative localization of the FBs. The navigation system facilitated precise surgical access and guided dissection while minimizing unnecessary tissue manipulation. In all cases, the FBs were accurately localized and successfully removed without significant intraoperative or postoperative complications. The procedures were completed without neurovascular injury or significant postoperative complications, and recovery was uncomplicated. The use of 3D‐IGNS enabled precise and minimally invasive surgical approaches, reducing the risk of injury to adjacent vital structures. These cases suggest that 3D‐IGNS is a safe and effective tool for managing complex CMF FBs, particularly in maxillofacial trauma cases where accurate localization is crucial to avoid injury to surrounding vital structures.

## 1. Introduction

Craniomaxillofacial (CMF) traumas, such as gunshots or penetrating FBs, usually occur as a result of accidental trauma, suicide attempts, human assault, blast injuries, and negligent occurrences. Gunshot wounds to the CMF are serious traumatic injuries that can lead to serious health issues such as soft tissue loss, bony destruction, infection, and functional issues [1]. Due to the presence of vital anatomical structures such as complicated blood vessels and nerve networks in the CMF region, precise detection of the spatial location and depth of foreign bodies, as well as surrounding structures, is critical because these structures are at risk of further intraoperative damage. FB retrieval is a difficult procedure due to injury risk to surrounding structures in the CMF region. When there are no fixed anatomical landmarks near FBs, the difficulty increases [2–4].

Surgical procedures in FB extraction are classified into two main categories: conventional and 3D‐IGNS‐based approaches. Although both approaches have relied on the preoperative images, conventional approaches could not provide any 3D visualization of delicate and complex structures from a single 2D image. Therefore, the retrieval of FBs in critical anatomical locations may lead to intraoperative bleeding, prolonged operative time, and damage to adjacent structures due to visual access limitations. So, they were found to be more invasive and might be inaccurate.

In recent years, there has been a considerable increase in the use of navigation systems in various surgical fields, especially in many maxillofacial surgery procedures [5]. Foreign body extraction, one of the most significant applications of navigation in trauma cases, follows the same procedures used in other navigation‐assisted surgeries. The general concept of surgical navigation is the intraoperative real‐time tracing of surgical tools by precise positioning systems and the indication of their location on patient‐specific images, which are reconstructed from preoperative images. Imaging, tracking, and registration are the key phases of navigation system–based surgery. The initial phase obtains diagnostic patient images preoperatively and uploads them to the navigation system′s software. It is worth noting that different imaging techniques, such as plain radiographs, computed tomography (CT), magnetic resonance imaging (MRI), or ultrasound, can be used to detect and locate FBs depending on their position and type. Among various imaging techniques, CT images are most commonly used for preoperative detection and intraoperative visualization of metallic foreign bodies in the CMF region for 3D‐IGNS [6]. In the second phase, the surgeon navigates the surgical tool to the target point intraoperatively and visualizes its location on preoperative images. To this end, a typical optical tracking system (OTS) and cameras can be used to follow surgical tools in stereophotogrammetric localization. Based on stereo visualization techniques, the pose of the tools in the camera′s working area is being tracked. In these systems, markers are retroreflective passive spheres based on transmitting or reflecting light fixed in a rigid alignment on the surgical navigation tool. OTS uses two cameras positioned in a fixed site to provide visual information from tracked surgical tools. The 3D location of the tools is determined based on the triangulation of the known geometric configuration of markers and the distance between charge‐coupled device (CCD) cameras (a CCD is a light‐sensitive integrated circuit that captures images by converting photons to electrons). The final phase is the process of patient‐to‐image registration, which correlates preoperative patient images with the patient′s real‐time spatial location during surgery, displayed on the navigation screen [7]. This minimally invasive surgical approach offers surgeons real‐time feedback on the position of foreign bodies and surrounding critical structures during procedures. It also enhances preoperative planning, scheduling, surgical precision, and postoperative evaluation.

## 2. The Report of Cases

This study presents several case reports of patients who underwent foreign body extraction using 3D‐IGNS at Sina Hospital in Tehran, Iran. The patients exhibited symptoms including pain, swelling, and functional limitations caused by foreign objects retained in the CMF area. Before the surgical intervention, written consent was acquired from each patient, and their clinical images were documented in this study. This clinical report used the Compo+ Parsiss surgical navigation system (Parseh Intelligent Surgical Systems Co., Tehran, Iran), an infrared‐based navigation technology, with a preoperative CT scan to locate and remove foreign bodies. Preoperatively, the patient′s CT scan was imported into the Compo+ software of the Parsiss surgical navigation system. In the Compo+ software, the FBs could be viewed through a virtual 3D model. During the surgical process, patients were placed supine, and their heads securely immobilized. Following the application of a general anesthetic as well as endotracheal intubation, the surgical site was carefully disinfected, and the dynamic reference frame was rigidly fixed on the patient′s skull. Then, surgical exploration through the specific incision (based on FB′s location) was carried out using the registered surgical tool to reach the precise position of the FB and confirm the real‐time registration between the operation position and the FB and important anatomical structures. Subsequently, in each case, the FB was precisely located and successfully removed with forceps without any significant complications. Screenshots were taken to document every step of the intraoperative navigation.

### 2.1. Case 1

**Figure 1:**
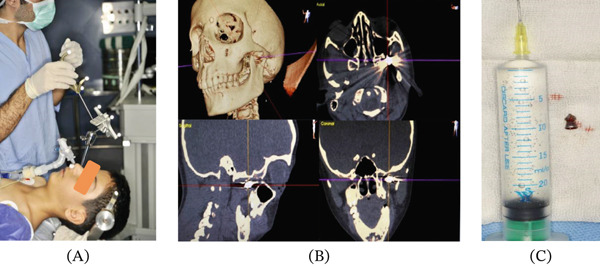
The surgical approach. (A) Reference frame and tool calibration for optical surgical navigation. (B) Intraoperative 3D CT scan with different views (axial, sagittal, and coronal) of FB location. (C) The extracted FB. CT, computed tomography; 3D, three‐dimensional; FB, foreign body.

An 8‐year‐old boy presented with a hunting pellet lodged in the infratemporal fossa, resulting from accidental play with a shotgun. The foreign body penetrated through the anterior wall of the right sinus and reached the temporal space (Figure 1A). While a large incision could be used to locate and extract the pellet, precise localization and removal can be challenging due to the pellet′s mobility and the presence of critical adjacent structures along the surgical path. In this case, the navigation system was employed to identify the pellet′s position, addressing these complexities accurately. Following patient and preoperative image registration, a 3 cm incision was made intraorally to confirm the pellet′s exact location (Figure 1B). The foreign body was then successfully identified and removed through the same incision path (Figure 1C). Notably, this approach minimized the risk of damage to the patient′s permanent teeth by preserving numerous tooth buds, and complications commonly associated with traditional methods were avoided.

### 2.2. Case 2

**Figure 2:**
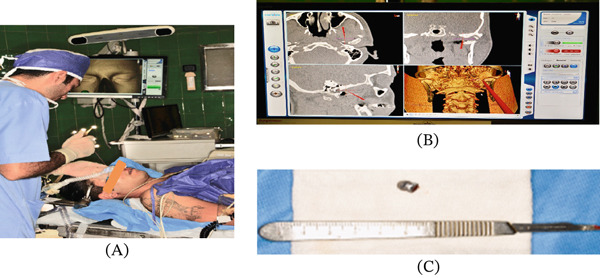
The approach of the surgery. (A) Navigation with infrared‐tracked tools: an infrared‐tracking camera and passive reflective spheres on the surgical instrument. (B) Patient‐image registrations lead to detecting the foreign body position using the navigation system. (C) The extracted foreign body.

Case 2 involved a 35‐year‐old male with an erosion of the mandibular condylar head caused by a metallic foreign body lodged in the medial aspect of the condylar head (Figure 2A). Using the surgical navigation system, we first detected the foreign body location accurately. Then, instead of an extraoral incision typically required for a condyle osteotomy, an intraoral incision in the anterior ramus was performed, and through dissection medial to the ramus, we successfully removed the foreign body through the same incision site (Figure 2B,C). According to the surgeon, given the complex anatomical area in this case, the mandibular condyle, the navigation‐based approach allowed for the use of minimally invasive intraoral access routes instead of conventional extraoral incisions. This approach helped improve cosmetic outcomes and reduce surgical complications. A crucial factor in the effectiveness of navigation is ensuring the foreign body remains completely fixed within the soft tissue; any movement or manipulation of soft tissue during surgery can reduce IGNS accuracy. Additionally, the interval between trauma and surgery is critical—if surgery is performed immediately post‐trauma, the desired precision may be compromised.

### 2.3. Case 3

**Figure 3:**
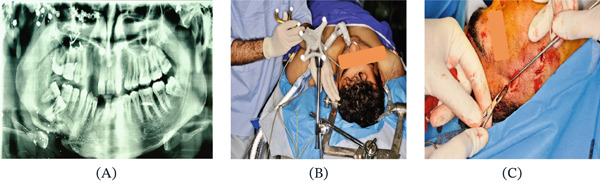
The surgical process. (A) OPG image of the patient indicates a large number of foreign bodies. (B) The registration processes. (C) Incision less than 0.5 cm, which is the same size as the foreign body.

The third case is perhaps the most intriguing: a 23‐year‐old male who sustained multiple gunshot wounds to the head and face from shotgun pellets. Due to severe upper facial injuries, many of the pellets were not visible on the orthopantomogram (OPG) image (Figure 3A). Using the navigation system, we successfully located and removed the penetrating foreign bodies through incisions smaller than 0.5 cm—approximately the same size as the pellets themselves (Figure 3B,C). The navigation system also allowed precise assessment of the depth of the foreign bodies. Given the large number of pellets and difficulty in accurately identifying their locations, conventional extraction methods could have required numerous facial incisions, compromising the aesthetic.

The parameters used to descriptively summarize the clinical and surgical outcomes of navigation‐assisted foreign body removal are presented in Table 1. It includes basic demographic information about each patient, foreign body location, surgical approach, incision length, operative time, and recorded intraoperative or postoperative complications.

**Table 1:** Summary of clinical and surgical outcomes.

Case	Age	FB location	Surgical approach	Incision length (cm)	Operative time (min)	Complications	Outcome
1	8	Infratemporal fossa	Intraoral	~3.0	< 15	None	Successful removal
2	35	Medial condylar head	Intraoral	~2.5	~45	None	Successful removal
3	23	Multiple facial pellets	Minimal incisions	< 0.5	~5 (per pellet)	None	Successful removal

## 3. Discussion

In general, FBs in the CMF region usually occur because of different traumatic events such as accidental trauma, suicide attempts, human assault, blast injuries, and negligent events. Because of the structural complexity of the CMF region, FBs pose a constant threat to adjacent tissues, and retained FBs in this area can lead to damage. Therefore, accurate and real‐time localization of FBs is essential for safe, minimally invasive, and successful surgery. In non‐navigated image‐based approaches, if the FBs are not close to a particular anatomical landmark, it may be difficult to precisely reconstruct this location in the patient′s physical anatomy [8]. Ultrasound is one of these approaches to remove foreign bodies. In their case study, Luo et al. [9] described how ultrasonic technology helped remove foreign bodies from the soft tissues of the oral and maxillofacial region. But despite being real‐time, the ultrasound is only appropriate for nonmetallic superficial foreign bodies located inside soft tissue. Otherwise, it is ineffective against foreign bodies located in hard tissues such as bone. Recently, 3D‐IGNS in FB extraction surgery has been applied for preoperative surgical planning and intraoperative FB observation. Therefore, registration of the preoperative images with the intraoperative anatomy enables the surgeon to perform real‐time, precise positioning of the FBs located in difficult access points [10, 11]. Such a property provides the identification of vital structures around FBs (such as delicate nerves and blood vessels) and safe FB extraction [12]. In 2015, Li et al. [13] presented a case of a 48‐year‐old female who was diagnosed with a foreign body near the mandibular canal. A head CT scan was performed with the open splint that was placed between the upper and lower dentition to stabilize the mandible at a specific open site in the mouth. After the registration process, the navigation surgery system was used to determine the accurate location and remove the object in a minimally invasive manner. In 2019, Sukegawa et al. [14] reported a case of a 78‐year‐old Japanese woman who had a foreign body in her maxillary bone. Because the FB was not adjacent to fixed anatomical landmarks and determining its precise position was extremely difficult, they used an optical surgical navigation system with a reference marker placed in a custom splint for registration. As a result, the foreign body was detected and removed in a less invasive manner. In 2023, Park [15] published the first application of a navigation system to remove a wooden foreign body. They described a 67‐year‐old male worker who presented to their hospital with a wooden foreign body on his right cheek. An emergency CT scan of the face indicated a foreign body that was broken into multiple pieces in the right zygomaticomaxillary bone in the unilateral nasal cavity. Additionally, a bony defect was caused by a penetrating injury from foreign bodies. They used a navigation system to remove these wooden foreign bodies safely in the paranasal areas, as well as reconstruct the bony defect. The possibility of foreign body displacement is an essential limitation when employing surgical navigation. Foreign body displacement during surgeries refers to their shifting or moving away from their intended positions within the surgical site. One of the key aspects to consider regarding foreign body displacement in surgeries is the time interval between trauma and surgery. The time interval plays a crucial role in the formation of a fibrous tissue mass surrounding each FB, facilitating their fixation. The other approaches are cone‐beam CT and C‐arm systems that are available intraoperatively to reproduce the new position of FBs in soft tissue [16].

The incorporation of basic quantitative surgical parameters (as shown in Table 1), including incision length and operative time, provides objective support for the minimally invasive nature of navigation‐assisted foreign body removal. In all presented cases, foreign bodies were successfully extracted without intraoperative or postoperative complications, and surgical access was achieved through limited incisions tailored precisely to the foreign body location. While no direct comparison with conventional techniques was performed, the observed outcomes suggest that navigation‐assisted surgery may reduce surgical exposure and tissue manipulation in complex CMF trauma cases. Despite the notable advantages of 3D‐IGNS, there are still limitations to it. As mentioned before, applying the navigation requires the fixation of the reference frame, which can be invasive and lead to injury, infection, scarring, and undesirable effects. Furthermore, the reference frame is acquired based on the premise that the system is fixed and not in constant change. So, the accuracy of the reference frame can be affected and decreased by any shift. Finally, navigation requires additional training for operators (especially for operators with the least experience with similar equipment) and extra costs for patients due to the complexity of the logistics and its relatively high price [17].

### 3.1. Clinical Novelty and Contribution of the Present Case Report

Although image‐guided navigation systems have previously been reported for foreign body removal in the CMF region, the present case report highlights several clinically relevant aspects that extend existing literature. First, the navigation‐assisted approach enabled the use of minimally invasive intraoral access routes instead of conventional extraoral incisions, particularly in anatomically complex regions such as the infratemporal fossa and the medial aspect of the mandibular condyle. This approach contributed to improved aesthetic outcomes and reduced surgical morbidity. Second, the application of navigation in a pediatric patient with deeply embedded metallic foreign bodies demonstrates the feasibility of preserving critical developing structures, such as permanent tooth buds, which is rarely emphasized in previous reports. Third, in cases involving multiple shotgun pellets, navigation‐assisted localization allowed accurate depth estimation and selective removal through subcentimeter incisions, a scenario in which conventional methods would likely require multiple exploratory incisions.

## 4. Conclusion

The first step in foreign body extraction surgery in the CMF region is to determine the accurate position of the FBs. Due to the existence of important anatomical structures in this area, even if the FBs themselves do not cause major problems, removing them can cause serious damage. Image‐guided navigation‐assisted surgery represents a feasible and promising approach for the localization and removal of deeply embedded foreign bodies in the CMF region. The presented cases demonstrate that navigation can facilitate minimally invasive surgical access, improve intraoperative orientation, and reduce the risk of injury to adjacent critical structures. Despite limitations such as potential foreign body displacement and the small number of cases, this technique shows considerable potential for complex trauma scenarios. The successful performance of navigation technology shows advantages such as less surgical invasion, improved preoperative surgical planning, timing, overall surgical accuracy, and postoperative evaluation. Further studies with larger cohorts and comparative designs are warranted to better define its clinical advantages over conventional approaches.

## Author Contributions

H.M.: conceptualization and design of the study and article drafting and critical revision. P.F.: conceptualization and design of the study, article drafting and critical revision, and final approval and guarantor of the manuscript. A.A.: conceptualization and design of the study, article drafting and critical revision, and final approval and guarantor of the manuscript. N.N.: final approval and guarantor of the manuscript. A.P.: conceptualization and design of the study, article drafting and critical revision, data acquisition, clinical/literature search, data analysis/interpretation, and final approval and guarantor of the manuscript.

## Funding

No funding was received for this manuscript.

## Consent

Patient consent is obtained.

## Conflicts of Interest

The authors declare no conflicts of interest.

## Data Availability

All data (photographs) used to support the findings of this report are included in this manuscript.
